# F29. HIGH-RISK SYMPTOMS FOR PSYCHOSIS IN ADOLESCENTS AND ITS RELATIONSHIP WITH FAMILY BURDEN

**DOI:** 10.1093/schbul/sby017.560

**Published:** 2018-04-01

**Authors:** Olga Puig-Navarro, Elena De la Serna, Jordina Tor, Anna Sintes, Gisela Sugranyes, Marina Redondo, Marta Pardo, Montse Dolz, Inmaculada Baeza

**Affiliations:** 1 Hospital Clinic of Barcelona; 2 Centro de Investigación Biomédica en Red de Salud Mental; 3 Hospital St Joan de Déu

## Abstract

**Background:**

High-risk symptoms for psychosis (HRS) and substantial functional impairment occurs early in the course of psychosis (Fusar-Poli et al., 2015). Many patients with HRS are adolescents who are still living at home and are highly reliant on their relatives for support. Objectives: (1) To compare the family burden of caregivers of adolescents with HRS with carers of an age and gender matched healthy control group (HC), (2) to examine the relationships between different family burden aspects and high-risk symptoms for psychosis in the HRS sample.

**Methods:**

Sample: 68 HRS subjects (15.3 ± 1.7 years, 66% females) and 42 HC subjects (15.5 ± 1.5 years, 66% females) from a prospective longitudinal study including help-seeking subjects who met HRS criteria (Child and Adolescent Psychiatry and Psychology departments of Hospital Clínic and Sant Joan de Déu, Barcelona, Spain). Inclusion criteria: age 10–17 years, meeting criteria for 1) attenuated positive or negative symptoms in the previous 12-months, 2) brief intermittent psychotic symptoms, 3) first or second degree relative with schizophrenia or schizotypical disorder plus impairment of functioning. Exclusion criteria: IQ<70, having a diagnosis of ASD. For HC subjects, exclusion criteria were having 1st or 2nd degree familiar with a psychotic disorder; a diagnosis of ASD and/or IQ<70. Instruments: the Semistructured Interview for Prodromal Syndromes and Scale of Prodromal Symptoms (SIPS/SOPS), the Hamilton Depression Scale and the Young Mania Scale for affective symptoms, a cognitive battery and the Caregiver Burden Inventory (CBI) which is a measure of family burden that has been validated in first-episode patients (McCleery et al., 2007). Caregivers’ responses are rated on a Likert scale from 0 (not at all descriptive) to 4 (very descriptive) and distributed in 5 factors: Time-Dependence Burden (T-Db), Developmental Burden (Db), Physical Burden (Pb), Social Burden (Sb), and Emotional Burden (Eb). High scores indicate greater perceived burden.

**Results:**

HRS and HC subjects did not significantly differ in age (t=0.68, p=0.497) and sex (X2=0.003, p=0.958). Intellectual quotient was higher in HC (mean=105.4 ± 11.28) than in HRS subjects (98.63 ± 14.27, t=2.53, p=0.013). Mean scores of high-risk symptoms in HRS subjects were higher than in HC subjects (t>-9.35, p<0.001): positive: 9.12 ± 4.76, negative:11.16 ± 5.49, disorganitzation:4.96 ± 3.03, general: 8.22 ± 3.83, and total symptoms:33.24 ± 12.59. HRS subjects had also higher scores in depressive (10.54 ± 7.54, t=-9.75, p<0.001) and manic symptoms (3.61 ± 4.53, t=-5.10, p<0.001). Caregivers of HRS subjects showed higher scores than caregivers of HC in all CBI subscales (t>-5.59, p<0.001; T-Db: 6.36 ± 5.01 vs 1.02 ± 1.60, Db: 7.42 ± 6.51 vs 0.45 ± 1.23, Pb: 7.00 ± 6.13 vs 0.58 ± 1.80, Sb: 4.77 ± 4.66 vs 0.64 ± 1.95, Eb: 4.86 ± 4.64 vs 0.93 ± 2.66). Time-Dependence burden reported by caregivers of HRS patients was significantly correlated with the SOPS total score (r=0.303, p=0.014) and with the negative SOPS subscale score (r=0.308, p=0.012). The relationship between negative SOPS symptoms and time-dependence burden remained after controlling for affective symptoms (F=5.07, p0.028) and intelligence quotient (F=7.27, p=0.009). This factor represents objective aspects of burden arising from demands on the caregiver’s time.

**Discussion:**

Caregivers of adolescents meeting criteria for HRS showed high perceived burden compared with caregivers of healthy adolescents. Time-dependence burden reported by caregivers was related to negative prodromal symptoms of HRS subjects. These findings highlighted that family burden occurs early in the course of psychosis. Acknowledgments: ISC-III/FIS, FEDER.

